# Simvastatin Induces Apoptosis in Medulloblastoma Brain Tumor Cells via Mevalonate Cascade Prenylation Substrates

**DOI:** 10.3390/cancers11070994

**Published:** 2019-07-17

**Authors:** Kimia Sheikholeslami, Annan Ali Sher, Sandhini Lockman, Daniel Kroft, Meysam Ganjibakhsh, Kazem Nejati-Koshki, Shahla Shojaei, Saeid Ghavami, Mojgan Rastegar

**Affiliations:** 1 Regenerative Medicine Program, Department of Biochemistry and Medical Genetics, Max Rady College of Medicine, Rady Faculty of Health Sciences, University of Manitoba, 745 Bannatyne Avenue, BMSB 627, Winnipeg, MB R3E 0J9, Canada; 2 Faculty of Medicine, University of Toronto, Toronto, ON M5S 1A1, Canada; 3 Department of Human Anatomy and Cell Sciences, Max Rady College of Medicine, Rady Faculty of Health Sciences, University of Manitoba, Winnipeg, MB R3E 0J9, Canada; 4 Research Institute of Oncology and Hematology, CancerCare Manitoba, Winnipeg, MB R3E 0V9, Canada

**Keywords:** medulloblastoma, brain tumor, apoptosis, mevalonate cascade inhibition, simvastatin

## Abstract

Medulloblastoma is a common pediatric brain tumor and one of the main types of solid cancers in children below the age of 10. Recently, cholesterol-lowering “statin” drugs have been highlighted for their possible anti-cancer effects. Clinically, statins are reported to have promising potential for consideration as an adjuvant therapy in different types of cancers. However, the anti-cancer effects of statins in medulloblastoma brain tumor cells are not currently well-defined. Here, we investigated the cell death mechanisms by which simvastatin mediates its effects on different human medulloblastoma cell lines. Simvastatin is a lipophilic drug that inhibits HMG-CoA reductase and has pleotropic effects. Inhibition of HMG-CoA reductase prevents the formation of essential downstream intermediates in the mevalonate cascade, such as farnesyl pyrophosphate (FPP) and gernaylgerany parophosphate (GGPP). These intermediates are involved in the activation pathway of small Rho GTPase proteins in different cell types. We observed that simvastatin significantly induces dose-dependent apoptosis in three different medulloblastoma brain tumor cell lines (Daoy, D283, and D341 cells). Our investigation shows that simvastatin-induced cell death is regulated via prenylation intermediates of the cholesterol metabolism pathway. Our results indicate that the induction of different caspases (caspase 3, 7, 8, and 9) depends on the nature of the medulloblastoma cell line. Western blot analysis shows that simvastatin leads to changes in the expression of regulator proteins involved in apoptosis, such as Bax, Bcl-2, and Bcl-xl. Taken together, our data suggests the potential application of a novel non-classical adjuvant therapy for medulloblastoma, through the regulation of protein prenylation intermediates that occurs via inhibition of the mevalonate pathway.

## 1. Introduction

Medulloblastoma is a common primary pediatric brain tumor that comprises about 15–20% of pediatric brain tumors. Medulloblastoma originates from the posterior part of the brain and cerebellum, which is an important brain structure with specific mature and immature cell types that are controlled at several levels [1,2,3,4]. In general, medulloblastoma can develop rather quickly and metastasize to different parts of the body via cerebrospinal fluid. The peak incidence of medulloblastoma ranges from 3–9 years of age in children, with 10% of cases in young infants. The survival rate for these patients varies with age, and as patients grow older, the survival rate decreases. There are four medulloblastoma subgroups, which have distinguishing features, and include: Wingless-INT (WNT), Sonic Hedgehog (SHH), subgroup 3, and subgroup 4 [5]. Current treatments for medulloblastoma include radiation, chemotherapy, and surgery [6,7,8,9]. However, radiation therapy has strict limitations, as it is usually contraindicated in young children, due to its adverse effects on brain development at a young age [10]. While chemotherapy is still a viable option, medulloblastoma has recently been shown to express resistance to many chemotherapeutic agents [11]. 

In the past few years, studies have highlighted the beneficial effects of statins in the treatment of different types of cancers. An important finding was presented in a large cohort of approximately 200,000 individuals that underlined the favourable impact of long-term statin usage in improving the survival rates in different cancer patients [12]. Statins inhibit 3-hydroxy-3-methylglutaryl-coenzyme A reductase (HMGCR), the rate-limiting enzyme in the mevalonate (Mev) pathway and cholesterol biosynthesis [13,14,15]. While cholesterol is the end product of the mevalonate cascade, the precursors of isoprenoids, which include farnesyl diphosphate (FPP) and geranylgeranyl diphosphate (GGPP), are important intermediates in the pathway [16,17]. Recent studies from independent scientific groups have revealed that statins induce apoptotic cell death in malignant and non-malignant cancer cells, including cancers of the breast [16], prostate [17], lung [18,19], human airway mesenchymal cells [20,21], and human atrial fibroblasts [22]. 

Here, we studied the cell death mechanisms by which simvastatin, a mevalonate cascade inhibitor, mediates its effects on three medulloblastoma cell lines. Simvastatin is an FDA-approved drug with minimal side effects, which passes the blood brain barrier (BBB) [23]. The three medulloblastoma cell lines include Daoy cells as a SHH subgroup, D283 cells belonging to the subgroup 3/4, and D341 cells from the subgroup 3 [5]. 

## 2. Results

### 2.1. Simvastatin Induces Cell Death in Medulloblastoma Cell Lines

**Figure 1:**
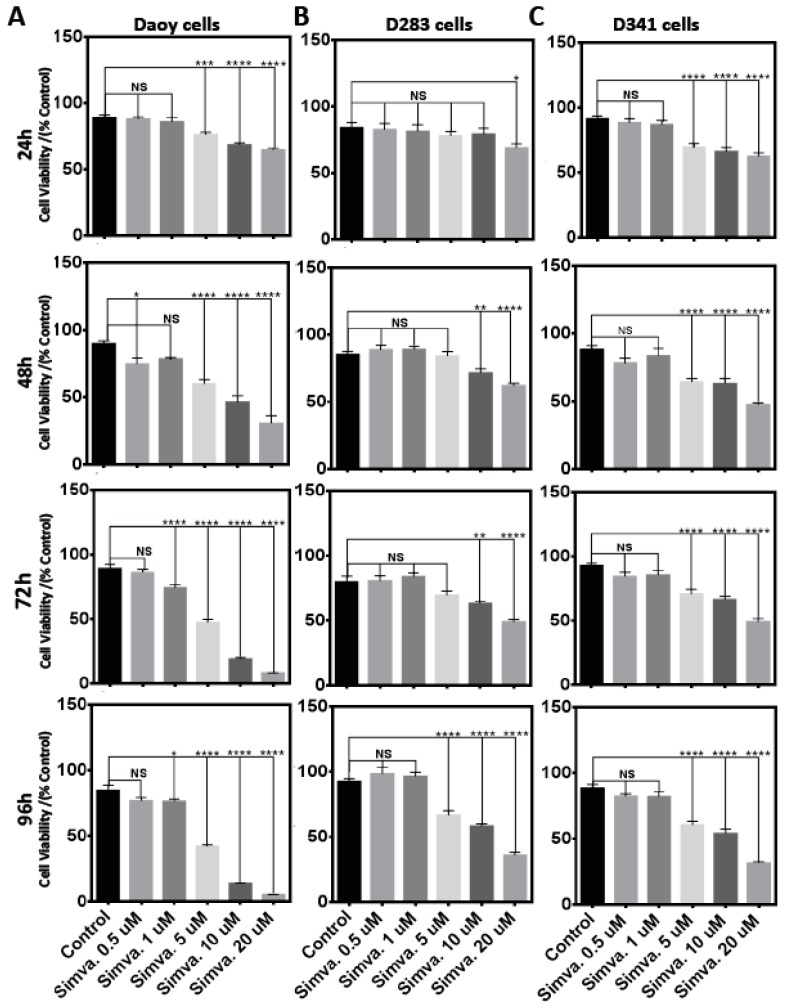
Simvastatin treatment induces significant cell death in medulloblastoma cells. Cell viability assays of Daoy (**A**), D283 (**B**), and D341 (**C**) cells, using dose-dependent analysis by 3-(4,5-Dimethylthiazol-2-yl)-2,5-diphenyltetrazolium bromide (MTT), performed every 24 h until 96 h (24 h, 48 h, 72 h, and 96 h). Medulloblastoma cells were treated with 0.5–20 µM simvastatin. Statistical significance is reported by one-way ANOVA using GraphPad Prism 7.0. The *p*-value is reported as **** *p* < 0.0001, *** *p* < 0.001, ** *p* < 0.01, or * *p* < 0.05. Data are expressed as means ±  SEM, and *n* = 15–20.

**Figure 2:**
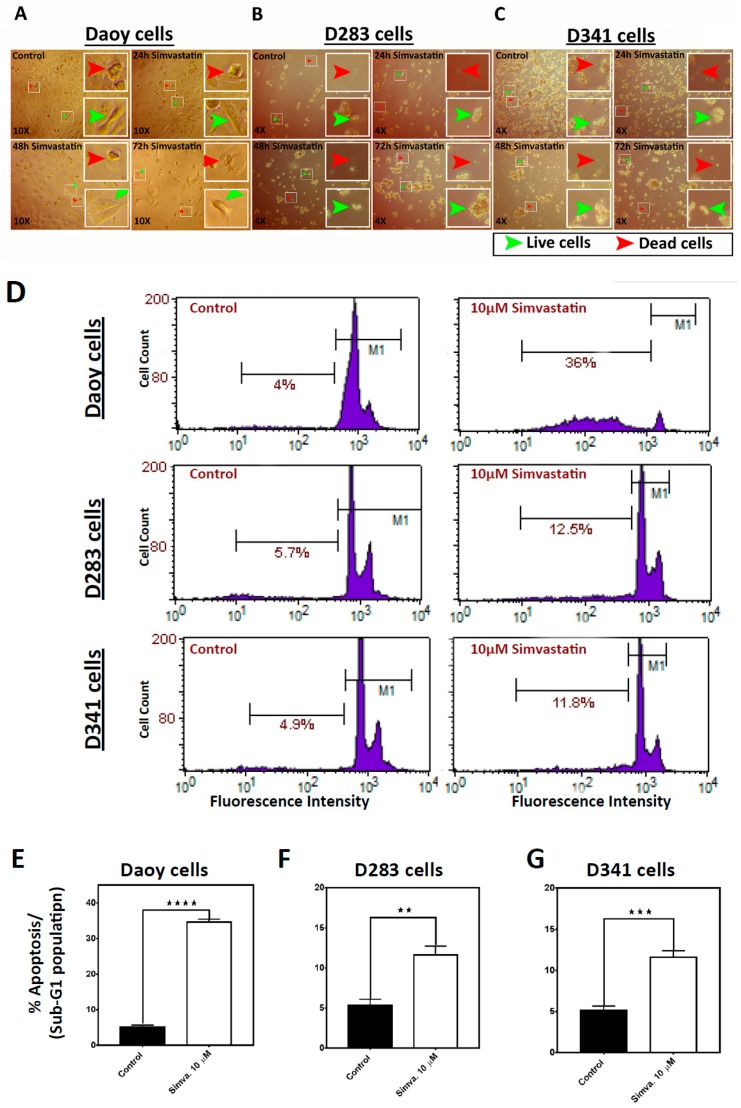
Simvastatin induces apoptosis in medulloblastoma cells. (**A**–**C**) The morphology of control and treated cells with 10 µM simvastatin is shown for the Daoy, D283, and D341 cells using a bright field microscope at 72 h. Green arrows indicate examples of live cells, and red arrows indicate examples of dead cells. (**D**) For flow cytometry, control and simvastatin-treated cells (10 µM) were collected at the 72 h time-point using standard cell collection protocol. Apoptotic cells were detected using Propidium Iodide (PI) Nicoletti flow cytometry and fluorescence-activated cell sorting (FACS) analysis. (**E**–**G**) Quantification of the sub-G1 population of results from part D, flow cytometry. There is an increased percentage of apoptotic cells in all tested cell lines (E: Daoy, F: D283, and G: D341). Statistical significance is reported by one-way ANOVA using GraphPad Prism 7.0. The *p*-value is reported as **** *p* < 0.0001, *** *p* < 0.001, or ** *p* < 0.01. Data are expressed as means ±  SEM, and with *n* = 3  ± SEM.

**Figure 3:**
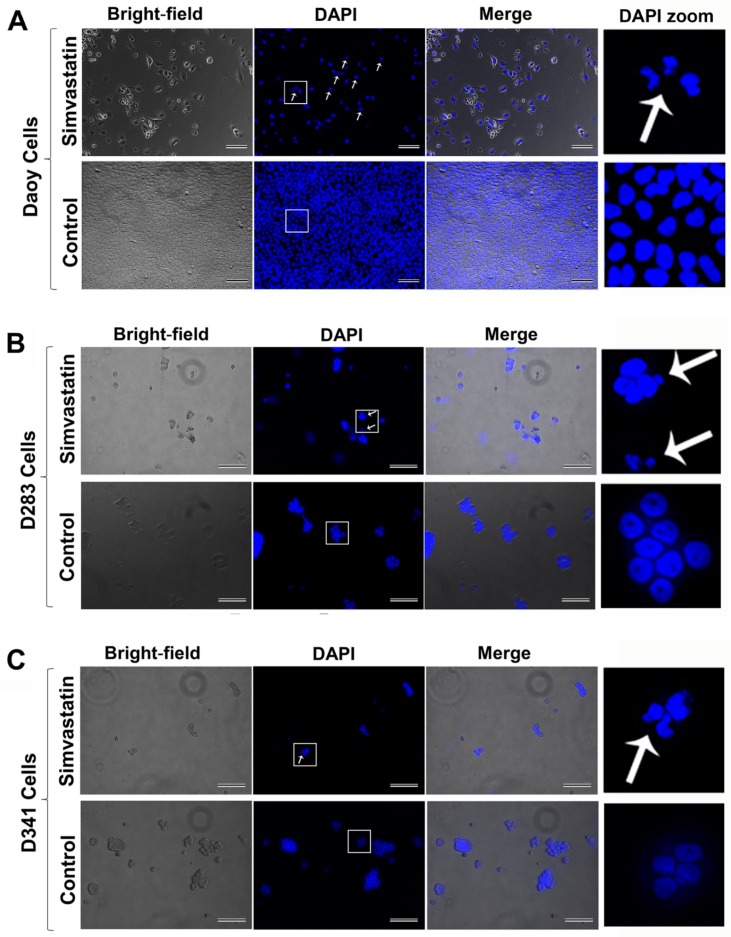
DAPI staining assay shows apoptosis in the nuclei of simvastatin-treated medulloblastoma cells. Daoy (**A**), D283 (**B**), and D341 cells (**C**) were cultured in 0.1% gelatin-coated plates and treated with 5 µM simvastatin, along with control non-treated cells for 72 h. Subsequently, cells were fixed, washed, and stained with DAPI at a 1 µg/mL final concentration. The simvastatin-treated cells showed condensed and fragmented nuclei (indicated by arrow). In the control non-treated cells, a normal morphology of nuclei was observed. Scale bar represents 50 µm.

To examine the possible cell death effects of simvastatin in medulloblastoma cells, Daoy, D283, and D341 cell lines were treated with 0.5–20 µM simvastatin at different time points [0–96 h (24 h, 48 h, 72 h, and 96 h)] (Figure 1A–C). Simvastatin induced significant cell death in Daoy cells [(24 h, *p* < 0.001 at 5 µM, and *p* < 0.0001 at ≥10 µM), (48 h, *p* < 0.05 at 0.5 µM, *p* < 0.0001 at ≥5 µM), (72 h, *p* < 0.0001 at concentrations ≥1 µM), (96 h, *p* < 0.05 at 1 µM, *p* < 0.0001 at ≥5µM)] (Figure 1A); D283 cells [(24 h, *p* < 0.05 at 20 µM), (48 h and 72 h, *p* < 0.01 at 10 µM, and *p* < 0.0001 at 20 µM), (96 h, *p* < 0.0001 at concentrations ≥5 µM)] (Figure 1B) and D341 cells [(24 h, 48 h, 72 h, and 96 h, *p* < 0.0001 at concentrations ≥5 µM)] (Figure 1C). The cellular morphology of control and simvastatin-treated cells was also monitored by bright-field microscopy (Figure 2A–C). In order to investigate whether simvastatin mediates its cell death effects through apoptosis, Daoy, D283, and D341 cells were treated with simvastatin (10 µM, 72 h) and analyzed by flow cytometry and fluorescence-activated cell sorting (FACS) flow cytometry (Figure 2D). Sub-G1 population analysis of the results indicated a significant increase in the percentage of apoptotic cells in Daoy (*p* < 0.0001) (Figure 2E), D283 (*p* < 0.01) (Figure 2F), and D341 (*p* < 0.001) cells (Figure 2G). Analysis of the nuclei morphology through DAPI (4′,6-diamidino-2-phenylindole) staining and fluorescence microscopy also showed that simvastatin-treated cells have condensed and fragmented nuclei, classifying them as apoptotic cells. In comparison, a normal nuclei morphology was observed in the control group of non-treated cells (Figure 3A–C). Taken together, our results show that simvastatin induces apoptosis in medulloblastoma cells.

### 2.2. Simvastatin Induces Caspase-Dependent Apoptosis in Medulloblastoma Cells

**Figure 4:**
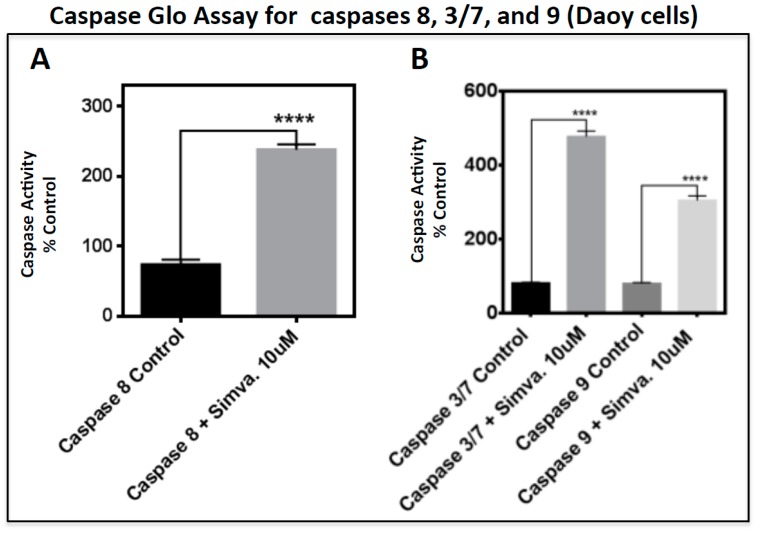

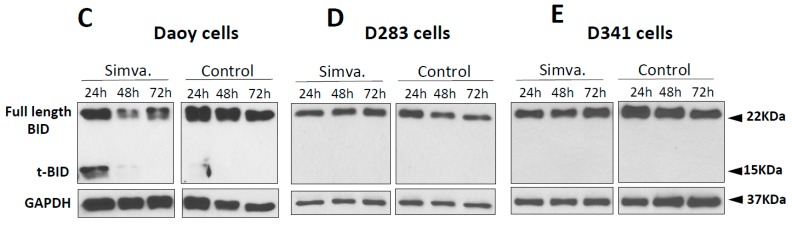
Simvastatin induces apoptosis via BID cleavage and caspase activities in Daoy cells. (**A**,**B**) Caspase activity in Daoy cells was measured by the Caspase Glow Assay kit at 48 h. The activity of caspase 8 is shown in A, and caspases 3/7 and caspase 9 are shown in B. Statistical significance is reported by unpaired t-test for caspase 8 activity and one-way ANOVA for caspase 3/7 and 9 using GraphPad Prism 7.0. The *p*-value is reported as **** *p* < 0.0001. Data are expressed as means ±  SEM, and *n* = 9–10. (**C**–**E**) For Western blots, Daoy, D283, and D341 cells were collected at 24 h, 48 h, and 72 h. GAPDH (Glyceraldehyde 3-Phosphate Dehydrogenase) detection of the same membrane was used as a loading control. For the continuous full length-image of Western blot signals in Figure 4C–E, please refer to Supplementary Figure S2.

To study the involvement of either intrinsic or extrinsic apoptotic pathways, we analyzed caspase 8 and BID (BH3 Interacting Domain Death Agonist) cleavage (proteins in the extrinsic apoptosis pathway), and caspases 3/7 and 9. The caspase 8 Glo assay was performed at 48 h (simvastatin 10 µM) to capture the peak activity of caspase 8. Our results showed a significant induction of caspase 8 activity in Daoy cells (*p* < 0.0001) (Figure 4A), yet no caspase 8 activation was detected in the other two cell lines (Supplementary Figure S1Aa,Ba). Caspase 8 induction in Daoy cells was also confirmed with the presence of truncated BID (t-BID) (Figure 4C). In Daoy cells, t-BID was detected by Western blot (WB) analysis at 24 h using an antibody that detects both BID and t-BID. These findings show that the extrinsic pathway is active in the early stage of apoptosis in Daoy cells and afterward, the whole apoptosis process relies on the intrinsic pathway. There was no significant change in the level of full-length BID in any of the simvastatin-treated cell lines (Figure 4C–E). Importantly, t-BID was only induced in Daoy cells, in the presence of caspase 8 activity (Figure 4A,C). Next, the activity of caspase 3/7 and caspase 9 was measured under the same experimental conditions as caspase 8. These results showed a significant induction of caspase 3/7 in all three tested cell lines (*p* < 0.0001) (Figure 4B, and Supplementary Figure S1Ab,Bb), yet caspase 9 was only induced in Daoy cells (*p* < 0.0001) (Figure 4B). We did not detect any significant increase in caspase 9 activity in D283 or D341 cells (Supplementary Figure S1Ab,Bb). Overall, our results suggest that simvastatin-induced cell death engages the apoptotic proteins BID, caspases 3/7, 8, and 9 via both intrinsic and extrinsic apoptotic pathways.

### 2.3. Simvastatin-Induced Cell Death in Medulloblastoma Cells Occurs via the Mevalonate Pathway and is Cell Line-Specific

**Figure 5:**
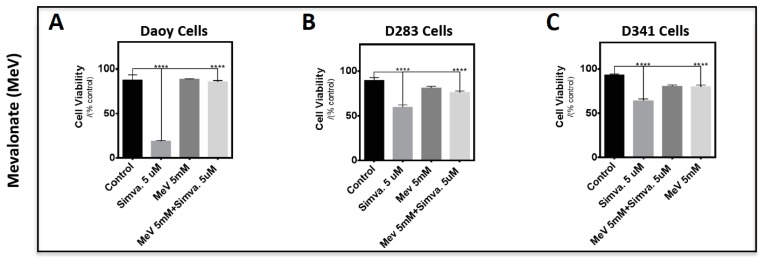

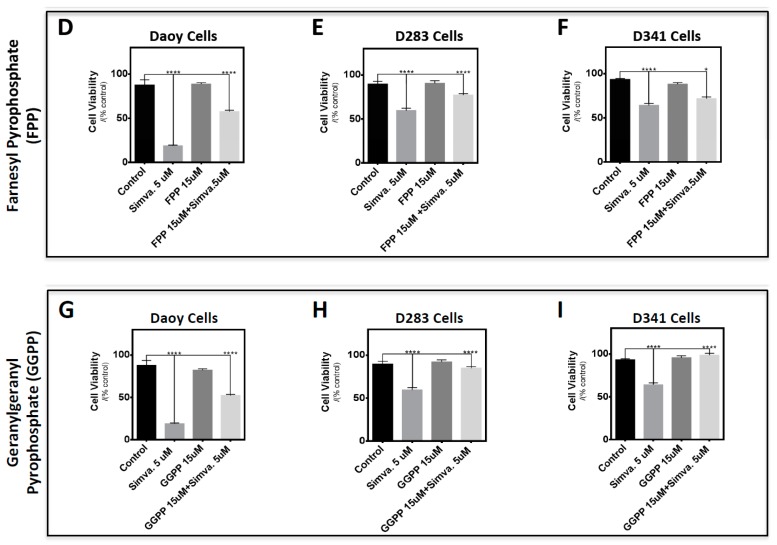
Mevalonate and isoprenoid intermediates rescue simvastatin-induced cell death via mevalonate cascade inhibition. Daoy (**A**,**D**,**G**), D283 (**B**,**E**,**H**), and D341 (**C**,**F**,**I**) cells were pre-treated with 5 mM mevalonate (**A**–**C**), 15 µM farnesyl pyrophosphate (FPP) (**D**–**F**), and 15 µM gernaylgerany parophosphate (GGPP) (**G**–**I**), followed by 5 µM simvastatin treatment. Cells were incubated for 96 h and cell viability was measured by the 3-(4,5-Dimethylthiazol-2-yl)-2,5-diphenyltetrazolium bromide (MTT) assay. Results show that MeV, FPP, and GGPP significantly reduce medulloblastoma cell death. Statistical significance was reported by one-way ANOVA using GraphPad Prism 7.0. *p*-value is reported as **** *p* < 0.0001 or * *p* < 0.05. Data are expressed as means ±  SEM, and *n* = 15.

In order to study the involvement of the cholesterol pathway in simvastatin-induced medulloblastoma cell death, Daoy, D283, and D341 cells were treated with MeV (5 mM), FPP (15 µM), or GGPP (15 µM), and after 3 h, this was followed by simvastatin co-treatment (5 µM for 96 h) (Figure 5A–I). The selected concentrations are based on our previous report [18]. Importantly, MeV blocked simvastatin-induced cell death in all three tested cell lines (Figure 5A–C, *p* < 0.0001). Next, we studied the involvement of FPP and GGPP in simvastatin-induced cell death. These results showed that similar to MEV, both FPP (Figure 5D–F, *p* < 0.0001) and GGPP (Figure 5G–I, *p* < 0.0001) blocked simvastatin-induced cell death in all three medulloblastoma cell lines. The rescue effect of FPP in D341 cells was also significant and was detected at *p* < 0.05 (Figure 5F). These findings highlight that simvastatin-induced cell death in medulloblastoma cells is dependent on the loss of mevalonate and isoprenoid intermediates. 

**Figure 6:**
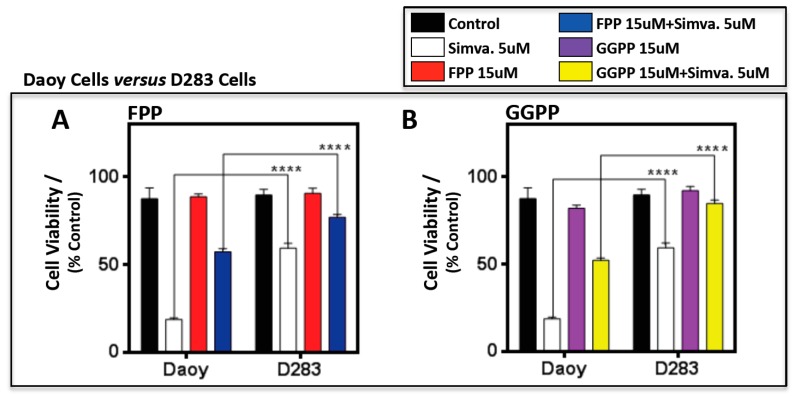

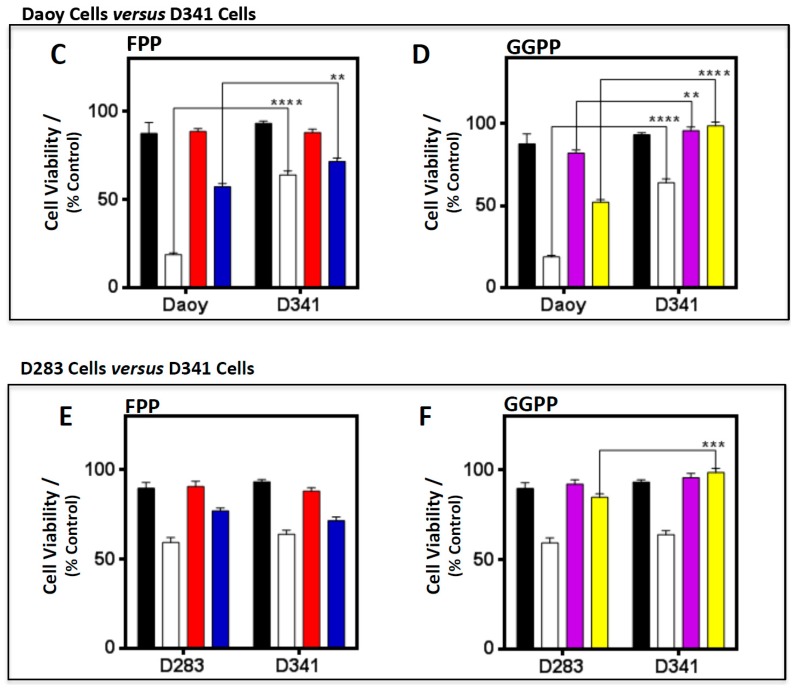
Farnesyl pyrophosphate (FPP) and gernaylgerany parophosphate (GGPP) have a cell line-specific response in medulloblastoma cell lines. Both FPP and GGPP levels were significantly reduced in Daoy cells compared to D283 (**A**,**B**) and D341 cells (**C**,**D**) when treated with simvastatin (5 µM) at 96 h. However, GGPP was observed to be significantly higher in D341 cells, when compared to Daoy cells, even under non-treatment conditions. Unlike Daoy *versus* D283 and D341, both FPP and GGPP levels remain unchanged between D283 and D341 under non-treatment conditions. Only GGPP was significantly reduced in D283 cells compared to D341 cells (**E**,**F**) when treated with simvastatin. Statistical significance was reported by two-way ANOVA using GraphPad Prism 6.0. *p*-value is reported as **** *p* < 0.0001, *** *p* < 0.001, or ** *p* < 0.01. Data are expressed as means ±  SEM, and *n* = 15.

A comparison of the results obtained from the three cell lines (Figure 6) showed that GGPP 100% reversed simvastatin-induced cell death in both D283 and D341 cells, while in Doay cells, GGPP could not rescue 100% of simvastatin-induced cell death. In addition, FPP and GGPP rescue is significantly higher in D283 and D341 cells compared to Doay cells (Figure 6A–D) (*p* < 0.0001, and *p* < 0.001). 

### 2.4. Simvastatin-Induced Cell Death is Associated with Altered Bcl-2 Family Protein Expression

**Figure 7:**
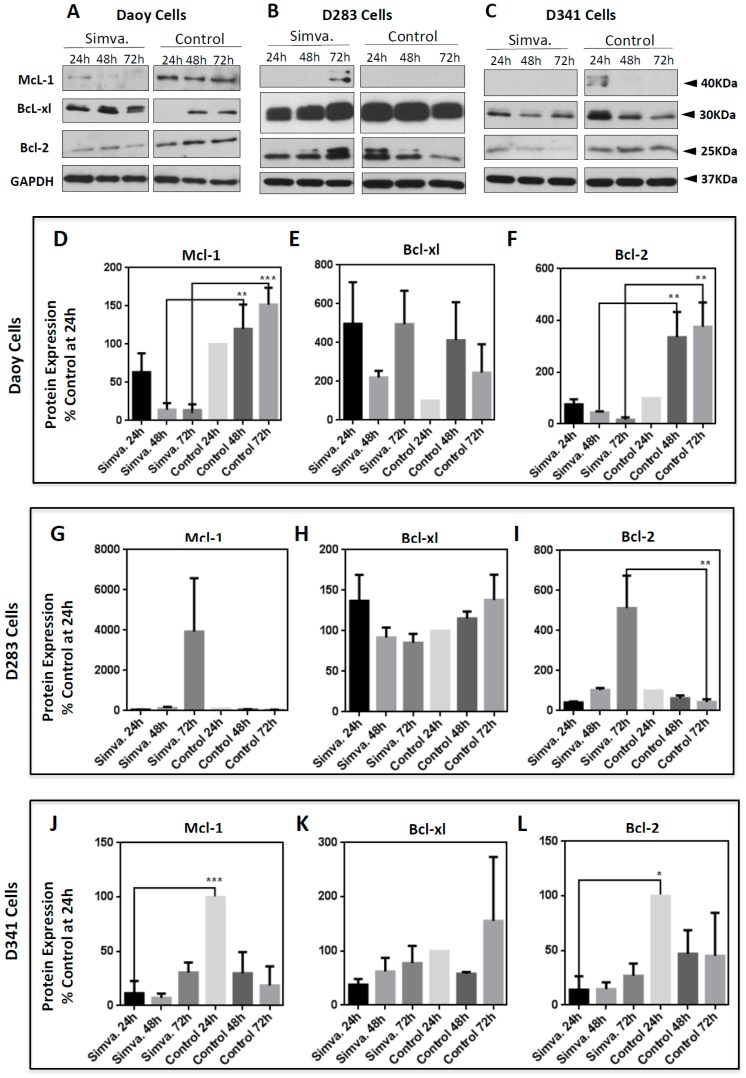
Simvastatin treatment alters the expression of anti-apoptotic proteins in the Bcl-2 family and is cell line-specific. Daoy (**A**,**D**–**F**), D283 (**B**,**G**–**I**), and D341 (**C**,**J**–**L**) cells were treated with 10 µM simvastatin and cells were collected every 24 h until 72 h, at which point Western blots for the anti-apoptotic proteins were performed. The anti-apoptotic proteins McL-1 (**A**–**D**,**G**,**J**), Bcl-xl (**A**–**E**,**H**,**K**), and Bcl-2 (**A**–**C**,**F**,**I**,**L**) were probed. Western blots were done to measure the expression of these anti-apoptotic proteins. GAPDH was used as the loading control. Western blot signals showing the expression of these pro-apoptotic proteins were quantified by ImageJ and Excel. Western blot signals were first normalized to the control at 24 h and these values were then normalized to their own GAPDH, which had also been normalized to its own control at 24 h. The simvastatin treatment values were then compared to their respective time-point control values and presented as a percentage. Data was collected in three independent biological experiments (*n* = 3) and expressed as means ± SEM. Statistical differences are evaluated by one-way ANOVA, using GraphPad Prism 6.0. *p*-value is reported as *** *p* < 0.001, ** *p* < 0.01, or * *p* < 0.05. For the continuous full length-image of Western blot signals in Figure 7A–C, please refer to Supplementary Figure S3.

To further elucidate simvastatin-induced cell death in medulloblastoma cells, we investigated the expression of anti- and pro-apoptotic proteins in the Bcl-2 family [Mcl-1, Bcl-xl, Bcl-2 (anti-apoptotic) and Bax (pro-apoptotic) at 24 h, 48 h, and 72 h]. Simvastatin (10 µM) treatment resulted in a significant decrease of the anti-apoptotic Bcl-2 protein in Daoy (48 h and 72 h, *p* < 0.01) (Figure 7A,F) and D341 cells (24 h, *p* < 0.05) (Figure 7C,L), yet not D283 cells, which showed a significant induction (72 h, *p* < 0.01) (Figure 7B,I). Similar to Bcl-2 expression in Daoy and D341 cells, the Mcl-1 protein was also significantly reduced by simvastatin treatment in Daoy cells (48 h, *p* < 0.01), (72 h, *p* < 0.001) (Figure 7A,D) and D341 cells (24 h, *p* < 0.001) (Figure 7C,J), but remained unchanged in D283 cells. We started the treatments at 50–60% confluency. As such, growing control cells at each time-point were compared to a previous time-point. It is therefore relevant that the protein expression changes in growing cultured cells, and our findings show this fact. Additionally, the Bcl-xl protein also remained unchanged in all three treated cell lines (Figure 7A–C,E,H,K). 

**Figure 8:**
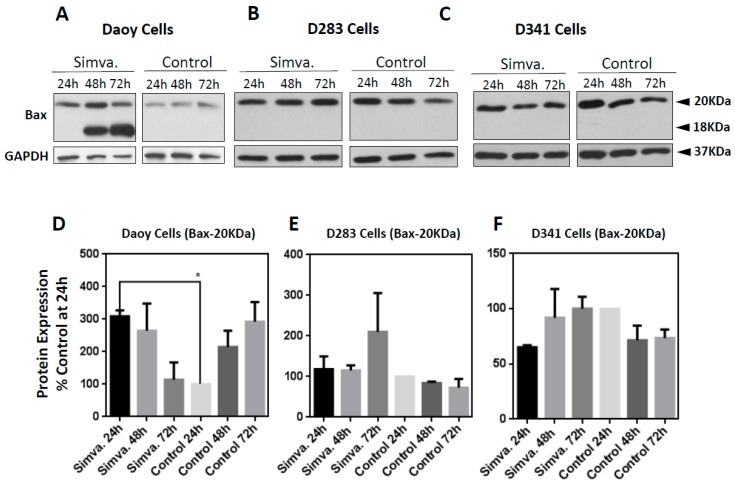
Simvastatin-induced expression of Bax is cell line-specific. Daoy (**A**,**D**), D283 (**B**,**E**), and D341 (**C**,**F**) cells were probed with the pro-apoptotic protein, Bax. Western blots were done to measure the expression of this pro-apoptotic protein. GAPDH was used as the loading control. Western blot signals showing the expression of these pro-apoptotic proteins were quantified by ImageJ and Excel. Western blot signals were first normalized to the control at 24 h and these values were then normalized to their own GAPDH, which had also been normalized to its own control at 24 h. The simvastatin treatment values were then compared to their respective time-point control values and presented as a percentage. Data was collected in three independent biological experiments (*n* = 3) and expressed as means ±  SEM. Statistical differences are evaluated by one-way ANOVA, using GraphPad Prism 6.0. *p*-value is reported as * *p* < 0.05. For the continuous full length-image of Western blot signals in Figure 8A–C, please refer to Supplementary Figure S4.

With regards to pro-apoptosis, simvastatin treatment (10 µM) resulted in a significant increase of Bax and cleaved Bax protein only in Daoy cells (Figure 8A,D) (24 h, *p* < 0.05), and not in D283 (Figure 8B,E) or D341 cells (Figure 8C,F). In summary, these findings suggest the involvement of pro- and anti-apoptotic proteins in the Bcl-2 family as a result of simvastatin treatment. 

## 3. Discussion

This study investigated the cell death mechanisms of mevalonate cascade inhibition (simvastatin) on medulloblastoma cells. Recently, the cytotoxic effects of statins have been studied in many different types of cancers. Accordingly, a bioinformatic study highlighted a potential therapy application of medulloblastoma cells by simvastatin [24]. However, there are no reports on the molecular mechanisms of the apoptosis-induced effects of simvastatin on medulloblastoma cells, representing a common pediatric brain tumor. Our results indicate that simvastatin induces cell death in three different medulloblastoma cell lines in a time- and dose-dependent manner. We show that simvastatin treatment significantly increases the number of apoptotic medulloblastoma cells, in comparison to the non-treated control cells. Interestingly, D283 and D341 cells showed a lower apoptotic response compared to Daoy cells, suggesting that different medulloblastoma subgroups may behave differently upon simvastatin treatment. Nonetheless, the induced cell death in D283 and D341 cells still remained quite significant when compared to their untreated control cells. Our results are in agreement with other *in vitro* studies that investigated the effect of statins on different types of cancers, including breast [25,26], prostate [27], pancreatic [28], and colon cancers [29]; osteosarcoma [30]; myelogenous leukemia [31]; and glioblastoma [23,32]. 

Our flow cytometry analysis revealed that simvastatin significantly increased the sub-G1 population of cells pointing towards an apoptotic cell death. In agreement with our results, Sanchez and Jang et al. reported statin-induced apoptotic cell death in MCF-7 breast cancer cells, although this partly occurred through a necrotic mechanism [26]. Caspase activation patterns confirmed our flow cytometry results, suggesting that simvastatin induced apoptotic cell death in each cell line through a different mechanism. In Daoy cells, which are derived from desmoplastic cerebellar medulloblastoma, considered to be less malignant, the extrinsic apoptotic pathway is involved. This was confirmed by caspase 8 activation, associated with the activation of executive caspases 3/7, which subsequently occurred. Activation of the extrinsic apoptotic pathway was further confirmed by WB analysis that showed increased levels of cleaved pro-apoptotic protein BID (truncated BID) by caspase 8 activity that subsequently resulted in the activation of an intrinsic apoptotic pathway, depicted by the increased activity of caspase 9 (in Daoy cells). However, in D283 and D341 cells, which are considered to represent more malignant medulloblastoma cells, apoptosis was not dependent on caspase 8 or caspase 9. This was further confirmed by WB analysis of the BID protein and the caspase-9 activation assay, where neither truncated BID nor caspase 9 activity was detected. It has been shown that statin-induced cell death in mesenchymal cells is independent of cytochrome C release [33]. Whether or not cytochrome C release is involved in the simvastatin-induced cell death in medulloblastoma cells warrants further investigations. It is also possible that caspae-3/7 activation in D341 and D283 cells is dependent on other mitochondrial protease enzymes; however, further investigations are required to address this hypothesis. Our rescue assay experiments showed that simvastatin-induced apoptosis in all tested medulloblastoma cell lines depends on the MeV pathway. Importantly, we observed that not only MeV (the first metabolite of the cholesterol pathway), but also FPP and GGPP (intermediate metabolites of the cholesterol pathway), rescued the simvastatin-induced apoptosis on the three tested medulloblastoma cell lines. These findings suggest that the inhibition of prenylated protein synthesis is a key mechanism to inducing apoptosis in these medulloblastoma cell lines. Isoprenoids, FPP, and GGPP, have a pivotal role in the processing of small G proteins, growth factor receptors, and nuclear lamins that are important in cell signaling and metabolism [34]. Accordingly, simvastatin induces cell death in cancer cells by inhibiting isoprenoid precursors, including FPP, and GGPP. This in turn leads to the loss of cholesterol from the cell membrane [35] and the anchorage of active small GTPase proteins, which are critically important for cell survival [36]. Therefore, we conclude that simvastatin induces cell death through inhibition of the mevalonate cascade, and prevents the formation of downstream intermediates in medulloblastoma cells. On the other hand, our results showed that GGPP fully reversed simvastatin-induced cell death in D341 and D283 cells, while it could not completely reverse the simvastatin cytotoxic effects in Daoy cells. These findings suggest that simvastatin-induced cell death may depend on the Rho, Rac, cdc42, and Rab small Rho GTPase protein in the D283 and D341 cells [37].

We further elucidated the cell death response in medulloblastoma cells treated with simvastatin, by investigating the percentage of apoptotic cells and the expression of pro-apoptotic marker proteins. In our results, we noted that the apoptotic cell death response induced by simvastatin is not only time-dependent, but also cell line-specific. Caspase-independent apoptosis is a known type of programmed cell death that has been frequently reported [38,39,40,41,42]. At 48 h, caspase 3/7 levels increase drastically in all three medulloblastoma cell lines, confirming that simvastatin-triggered cell death in D283 and D341 cells is independent of caspase 8 and 9. However, the simvastatin-induced cell death involves the intrinsic apoptosis pathway, which is dependent on the activation of caspase 3/7 [43,44,45]. 

While simvastatin induced significant cell death in all three medulloblastoma cell lines, there were variations in the expression of Bcl-2 pro- and anti-apoptotic proteins after simvastatin treatment. Mcl-1 is an anti-apoptotic protein that regulates apoptosis [46] and its downregulation triggers the apoptotic cell death [47,48]. Here, we showed that simvastatin decreased Mcl-1 expression in Daoy and D341, without being changed in D283 medulloblastoma cells. This finding is in agreement with the results reported by Demyanets et al. on the effect of statins on cardiac myocytes [49]. In the presence of simvastatin, anti-apoptotic protein Bcl-2 exhibits a drastically decreased expression in both Daoy and D341 cells, with the opposite trend in D283 cells, where it was increased. Increased expression of Bcl-2 by statins has also been reported in SH-SY5Y neuroblastoma cells [50]. It appears that the effect of statin on the Bcl-2 level is also cell line-dependent, as on vascular smooth muscle cells, statin treatment resulted in the downregulation of Bcl-2 [51]. Additionally, another *in vitro* study on different types of solid tumors, i.e., hepatocellular carcinoma, breast cancer, gastric cancer, and non-small lung cancer cells, has reported the decreased levels of Bcl-2 expression in response to statins, supporting the potential of cell type-specific effects of statin [52]. In desmoplastic cerebellar medulloblastoma Daoy cells, Bcl-2 shows the opposite response to the intrinsic pro-apoptotic protein Bax, which displays increased expression, and decreases over time compared to control cells. In malignant D283 and D341 medulloblastoma cells, expression of the pro-apoptotic protein Bax shows no significant change compared to control cells. This suggests a high basal level of this protein in these cell lines. Recent studies have identified Bcl-2 as a modulator of apoptosis, which has opened up novel therapeutic treatment strategies for cancer [53]. Under physiological conditions, Bcl-2 expression is high enough to bind and suppress Bax activity [54,55]. This interaction prevents its pro-apoptotic activity in cells. However, in our study, we observed that, in D283 cells, Bcl-2 behaves more like an apoptotic protein in a similar fashion to Bax. Our data indicates that Bcl-xl protein expression remains unchanged in all three cell lines. Our results indicate that the pro-and anti-apoptotic protein expression in Daoy cells behaves differently compared to D283 and D341 cells. The varying response of pro- and anti-apoptotic proteins in these three tested medulloblastoma cell lines implies that simvastatin may have a sub-group-specific and time-dependent effect. Taken together, this suggests that simvastatin might be more effective on desmoplastic cerebellar medulloblastoma than malignant medulloblastoma, which is a key consideration in medulloblastoma treatment. 

## 4. Materials and Methods

### 4.1. Reagents

Culture media, including the minimum essential media (MEM), penicillin-streptomycin-glutamine, sodium pyruvate, fetal bovine serum (FBS), and DAPI, were purchased from Thermofisher Scientific. The following reagents were acquired from Sigma (Mendota Heights, MN, USA): secondary anti-rabbit antibody, simvastatin, MeV, FPP, GGPP, cholesterol, and MTT. Rabbit anti-human Bax, Bcl-2, Bcl-xl, and Mcl-1 were purchased from Cell Signaling. Anti-mouse monoclonal GAPDH was purchased from Santa Cruz Biotechnology, and Casapase-Glo^®^-3/7, Caspase-Glo^®^-8, and Caspase-Glo^®^-9 assays were obtained from Promega (Madison, WI, USA). 

### 4.2. Cell Culture and Cell Viability Assays (MTT)

The following human medulloblastoma cell lines: Daoy (ATCC- HTB-186™), D283 (ATCC-HTB-185TM), and D341 (ATCC-HTB-187TM), were directly purchased from ATCC. Daoy cells were cultured in MEM (Invitrogen, Carlsbad, CA, USA) supplemented with 10% fetal bovine serum, 1% penicillin/streptomycin and glutamine, and 1% sodium pyruvate. D283 and D341 cells were cultured in the same media, but without sodium pyruvate. MTT assays were conducted based on a previously established protocol [33]. We seeded 2000 cells/well in 96-well plates, and allowed cells to grow to ~50% confluency. Cells were then treated with 0.5, 1, 5, 10, and 20 µM simvastatin. Cell viability assays were measured every 24 h, until the 96 h time-point. To measure cell viability, 20 µL of MTT dye (Sigma) was added to each well and the plate was then incubated at 37 °C for 3 h, and MTT studies were performed as reported previously [55,56,57]. The media and reagent were carefully removed from each well (for non-adherent D283 and D341 cells, the 96-well plate was centrifuged at 1500× *g* for 5 min at 4 °C), and Dimethyl Sulfoxide (DMSO) was then added to the wells. The content of each well was properly mixed by pipetting, followed by measuring the absorbance of the 96-well plates at 570 nm.

### 4.3. Immunofluorescence

DAPI staining was performed to show apoptosis in the nuclei of simvastatin-treated medulloblastoma cells. The cells were cultured in 0.1% gelatin-coated 24-well plates and simvastatin was added to the wells at a 5 µM concentration for 72 h. The cells were then fixed with 100% methanol and washed with phosphate saline buffer (PBS). Then, DAPI was added to the cells at a concentration of 1 µg/mL for 5 min. The nuclei of the simvastatin-treated cells and non-treated cells were analyzed by a Zeiss fluorescence microscope.

### 4.4. Rescue Treatments

Rescue experiments were done according to previously reported protocols [27]. Cells were seeded and grown in 96-well plates at a density of 2000 cells/well, up to 50% confluence. Cells were initially treated with 5 mM MeV, 15 µM FPP, and 15 µM GGPP, and were incubated at 37 °C for 3 h. A parallel set of controls were treated individually either with 5 mM MeV, 15 µM FPP, or 15 µM GGPP. For co-treatment, the cells treated with MeV, FPP, or GGPP were then treated with 5 µM simvastatin and incubated at 37 °C for 96 h. The 96 h incubation period allowed for the exhaustion of all residual cholesterol intermediates present within the cell and incorporation of the cholesterol intermediates added externally to the plates. Cell viability was then measured using the MTT assay after 96 h, as described in the previous section.

### 4.5. Nicoletti Apoptosis Assay

The apoptosis assay was done based on our modified Nicoletti PI flow cytometry assay [34]. Cells were seeded in 12-well plates with a concentration of 50,000 cells/well, and grown until 50% confluence. Cells were treated with 10 µM simvastatin, along with control non-treated cells, and were incubated at 37 °C for 72 h and then collected. Adherent Daoy cells were detached using EDTA (ethylenediaminetetraacetic acid) buffer, and non-adherent D283 and D341 cells were collected by pipetting. Cells were washed twice with PBS and re-suspended in a hypotonic PI lysis buffer. The cell nuclei were then incubated at 37 °C for 30 min and subjected to flow cytometry analysis. Apoptotic cell nuclei were gated, which were observed on the left side of the G1 peak with hypo-diploid DNA.

### 4.6. Luminometric Caspase Assays

Caspase 3/7, 9, and 8 activities were measured by a luminometric caspase assay [(Caspase-Glo 9 Substrate: G816A), (Caspase-Glo 8 Substrate: G815A), and (Caspase-Glo 3/7 Substrate: G811A)] in simvastatin-treated cells [35]. Cells were seeded in 96-well plates at 2000 cells/well and grown until about 50% confluence. Cells were treated with 10 µM simvastatin, along with non-treated controls. We incubated the 96-well plates at 37 °C for 48 h. Next, MG-inhibitor was centrifuged at 1000 rpm for 30 s at 4 °C and added to the caspase buffer. This mixture of caspase buffer + MG-inhibitor was added to the caspase substrate and mixed. From each well, 100 µL of media was removed, followed by the addition of 100 µL of the prepared caspase reagents. Plates were then covered with aluminum foil and shaken for 20 min at room temperature. From these plates, 150 µL of media was removed from each well, and transferred to a white 96-well plate. The absorbance of this plate was measured using a luminometric plate reader.

### 4.7. Western Blots

We performed Western blots to detect Mcl-1, Bcl-xl, Bcl-2, and Bax in the total protein extracts of Daoy, D283, and D341 cells. GAPDH was used as the loading control. Total cell proteins were extracted by lysing in a buffer containing 50 mM Tris-HCl (pH 8.0), NP-40 (1.0%), NaCl (150 mM), and 0.5% of the Roche protease inhibitor cocktail. This was followed by 8 min of centrifugation (10,000× *g*), and the quantification of total cell protein lysates by the Bradford protein assay, as previously reported [58]. We used denaturing SDS-PAGE to run protein samples, and transferred the samples to PVDF (Polyvinylidene Difluoride) membranes. These membranes were then blocked with 3% milk-Tween 20, and incubated overnight with primary antibody [Cell Signalling antibodies: Bax (2774S), Bid (2002S), Mcl-1 (4572S), Bcl-xl (2762S); ThermoFisher Scientific: Bcl-2 (MA5-11757); and Santa Cruz Biotechnology: GAPDH (sc-47724)] at 4 °C. After three washes, membranes were incubated with HRP-conjugated secondary antibody [Sigma-Aldrich: Anti-Rabbit IgG (A6154), and Jackson ImmunoResearch: Peroxidase-conjugated affinpure Goat anti-Mouse IgG, Light chain specific (115-035-174)] (1 h, room temperature). The enhanced chemiluminescence (ECL) kit from Amersham-Pharmacia Biotech was used for blot development, as previously reported [59,60].

### 4.8. Microscopic Imaging of Live Cells

Cells with 50% confluency in 10 cm plates were treated with 10 µM simvastatin, incubated at 37 °C for 96 h, and imaged under a Zeiss microscope at 4× and 10× by a Nikon Camera, as previously reported [61,62].

### 4.9. Statistical Analysis

Results from different experiments are presented as M ± SEM (means ± standard error of mean) and statistical significances were calculated by one-way ANOVA, unpaired t-test, or two-way Anova, by using GraphPad Prism 6.0 or 7.0 software. Results with a *p*-value  < 0.05 were considered statistically significant, as previously reported [63,64,65,66]. For reproducibility, MTT experiments were conducted in five replicates, with a minimum of three independent experiments. All WB experiments included three independent biological replicates. All WB signals were quantified by ImageJ and processed by Excel. The Western blot signals were first normalized to their own control sample (i.e., control at 24 h) and then normalized to their own GAPDH loading control, which was first normalized to its own control sample at 24 h. The simvastatin treatment values were then compared to their respective time-point control values and represented as a percentage expression value. Statistical analysis was conducted as described above and reported previously [21,40,41,42,43,44].

## 5. Conclusions

Mevalonate pathway inhibition by simvastatin induced significant apoptotic cell death in SHH and group 3/4 medulloblastoma cell lines, revealing prenylation of the cholesterol-related pathways. Cell viability, FACS, caspase glow assays, and WB analysis highlighted the significant cytotoxic effects of simvastatin, resulting in the up-regulation of pro-apoptotic proteins. Moreover, medulloblastoma cell rescue by MeV, FPP, and GGPP highlighted the importance of downstream mevalonate cascade intermediates in medulloblastoma development and progression. This study suggests a non-classical therapeutic avenue for medulloblastoma treatment and paves the path for simvastatin to be potentially used in combination with other classical anti-cancer drugs, as a more efficient and effective adjuvant chemotherapy agent.

## Supplementary Materials

The following are available online at https://www.mdpi.com/2072-6694/11/7/994/s1: Figure S1: Caspase activities in D283 and D341 cells, Figure S2: The continuous full length-image of Western blot signals shown in Figure 4C–E. For description of the samples, please refer to Figure legends (4C–E), Figure S3: The continuous full length-image of Western blot signals shown in Figure 7A–C. For description of the samples, please refer to Figure legends (7A–C). Please note that for some Western blots, an additional time 0 has been tested. The time 0 (0 h) refers to the collected cells at the starting time for simvastatin treatments, Figure S4: The continuous full length-image of Western blot signals shown in Figure 8A–C. For description of the samples, please refer to Figure legends (8A–C). Please note that for some Western blots, an additional time 0 has been tested. The time 0 (0 h) refers to the collected cells at the starting time for simvastatin treatments.

## Abbreviations

Acetyl Co-A: Acetyl Coenzyme-A; BBB: Blood Brain Barrier; BID: BH3 Interacting Domain Death Agonist); DAPI: 4′,6-diamidino-2-phenylindole; DMSO: Dimethyl Sulfoxide; EDTA: ethylenediaminetetraacetic acid; FACS: Flow Cytometry and Fluorescence-Activated Cell Sorting; FBS: Fetal Bovine Serum; FPP: Farnesyl Pyrophosphate; GAPDH: Glyceraldehyde 3-Phosphate Dehydrogenase; GGPP: Geranylgeranyl Pyrophosphate; HMGCR: 3-hydroxy-3-methylglutaryl-coenzyme A reductase; MeV: Mevalonate; MTT: 3-(4,5-Dimethylthiazol-2-yl)-2,5-diphenyltetrazolium bromide; PBS: Phosphate Buffered Saline; PI: Propidium Iodide; PVDF: Polyvinylidene Difluoride; SHH: Sonic Hedgehog; t-BID: truncated BID; WB: Western blot; Wingless-INT: WNT.
