# Supplementary material for: Simvastatin Induces Apoptosis in Medulloblastoma Brain Tumor Cells via Mevalonate Cascade Prenylation Substrates
Source: Cancers (Basel). 2019 Jul 17;11(7):994. doi: 10.3390/cancers11070994 (PMC6678292; doi:10.3390/cancers11070994)
Supplement: Supplementary file 1 [file cancers-11-00994-s001.pdf]

Supplementary Materials: Simvastatin Induces Apoptosis in Medulloblastoma Brain Tumor Cells via Mevalonate Cascade Prenylation Substrates

Kimia Sheikholeslami, Annan Ali Sher, Sandhini Lockman, Daniel Kroft, Meysam Ganjibakhsh, Kazem Nejati-Koshki, Shahla Shojaei, Saeid Ghavami, and Mojgan Rastegar

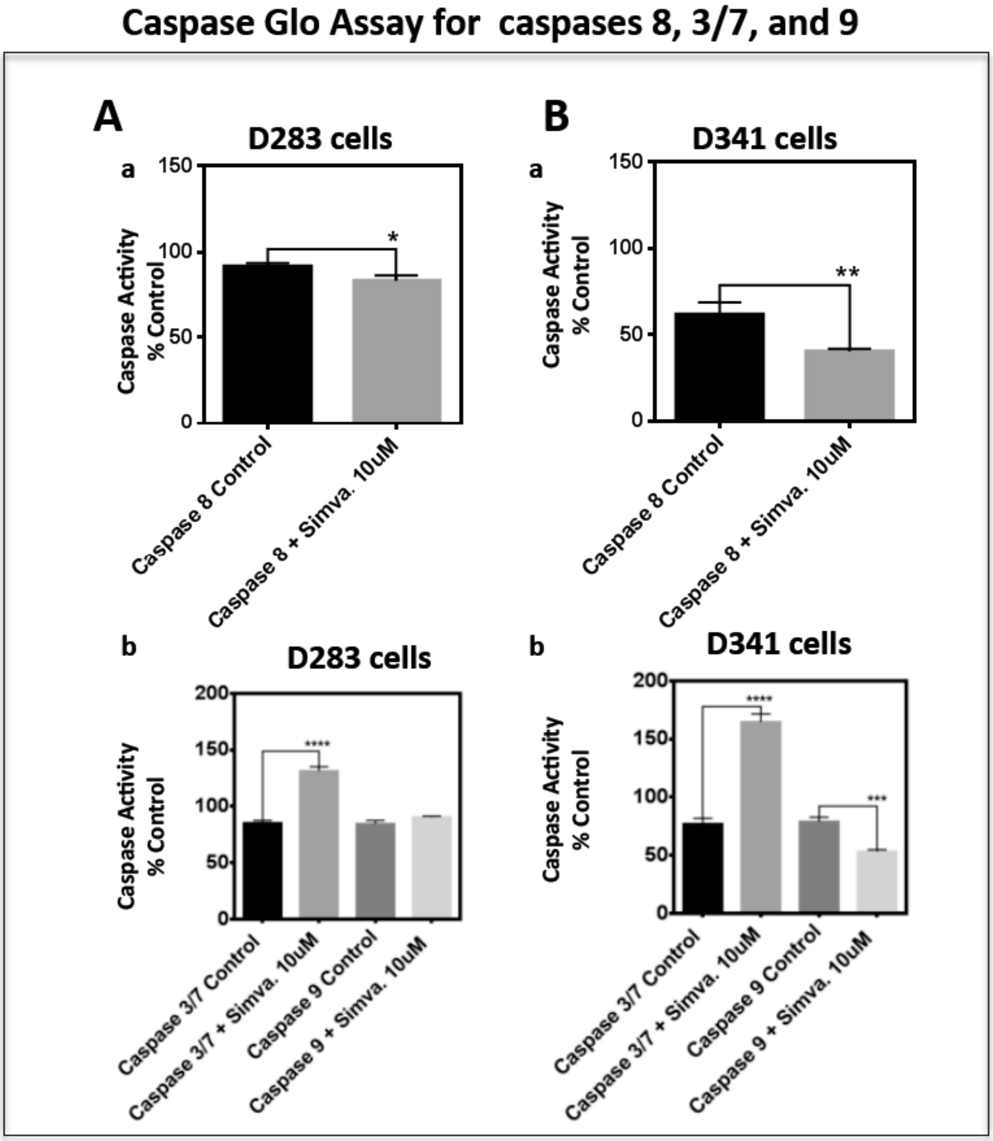

Figure S1. Caspase activities in D283 and D341 cells. Caspase activity was measured by the Caspase Glow Assay kit at 48 h, following instructions outlined in the kit manual, in D283 cells (**A**) and D341 cells (**B**). Caspase 8 activity is shown in Aa and Ba, and caspases 3/7 activities are shown in Ab and Bb. Statistical significance is reported by unpaired t-test for caspase 8 activity and one-way ANOVA for caspase 3/7 and 9 activity, using GraphPad Prism 7.0. The *p*-value is reported as **** $p < 0.0001$, *** $p < 0.001$, ** $p < 0.01$, or * $p < 0.05$. Data are expressed as means ± SEM, and $n = 10$.

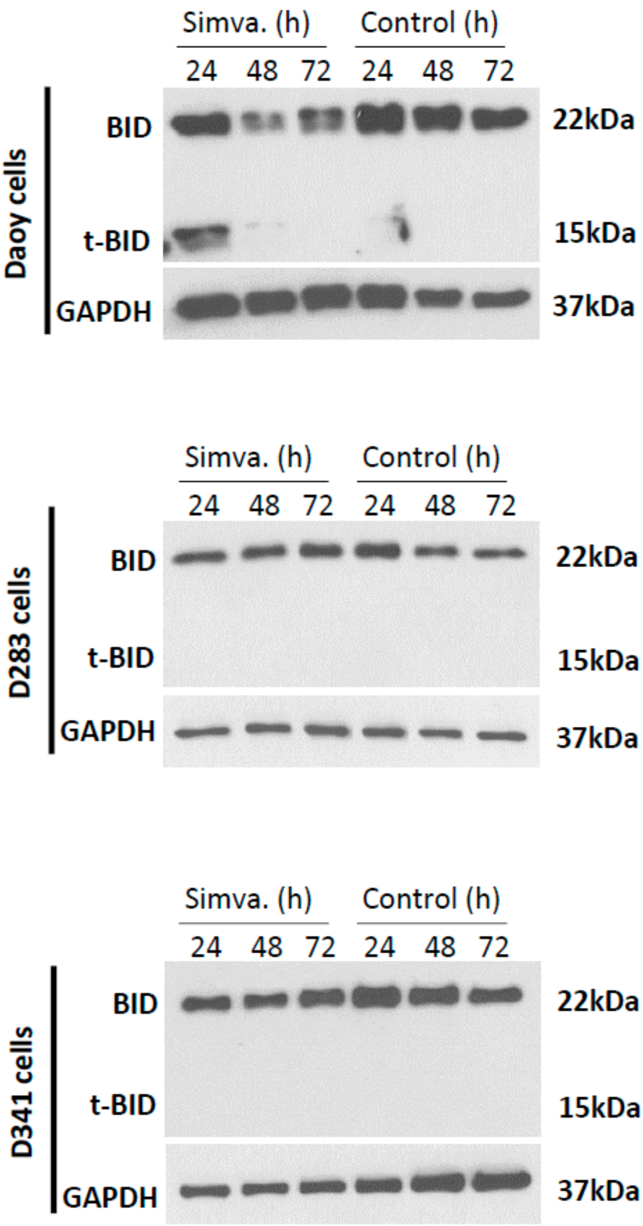

Figure S2. The continuous full length-image of Western blot signals shown in Figure 4C–E. For description of the samples, please refer to Figure legends (4C–E).

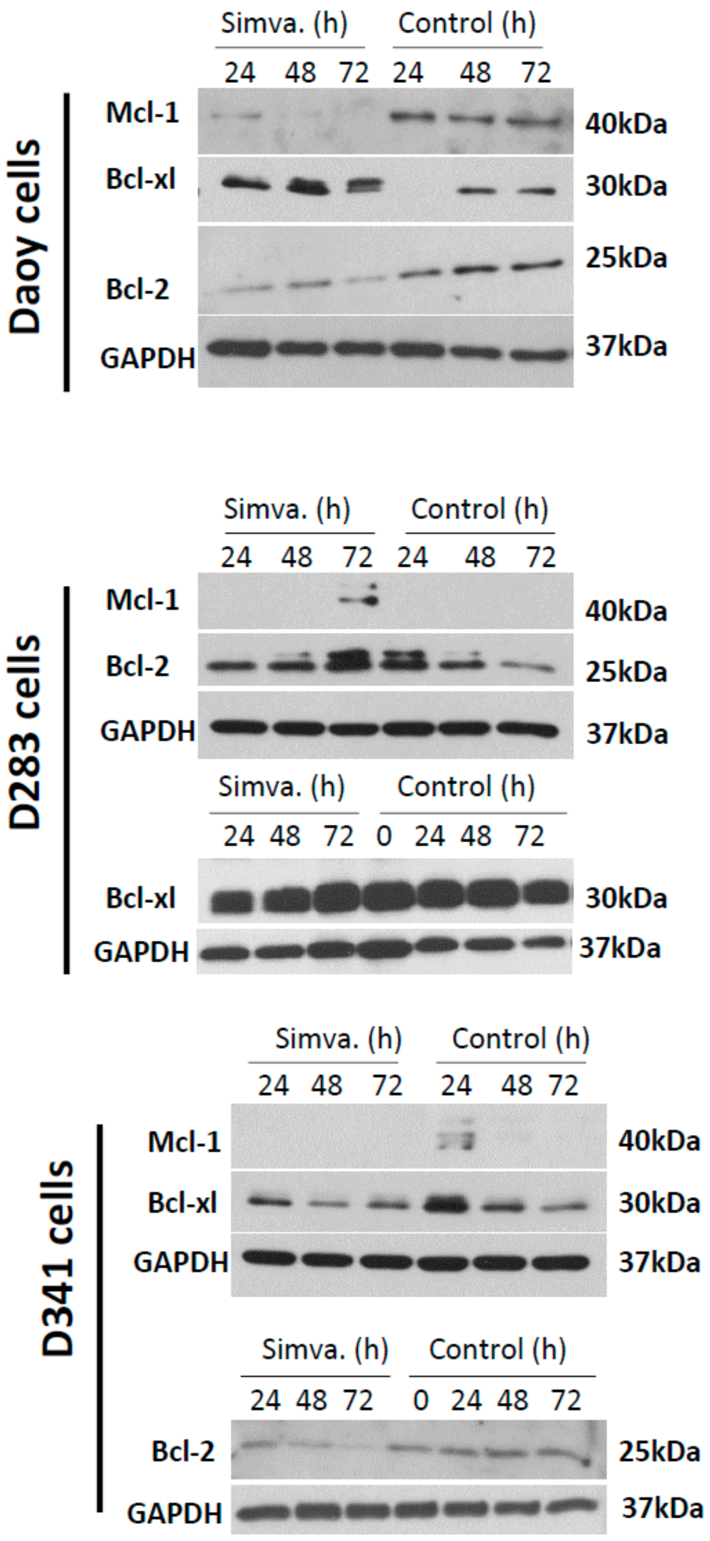

Figure S3. The continuous full length-image of Western blot signals shown in Figure 7A–C. For description of the samples, please refer to Figure legends (7A–C). Please note that for some Western blots, an additional time-point 0 has been tested. The time 0 (0 h) refers to the collected cells at the starting time for simvastatin treatments.

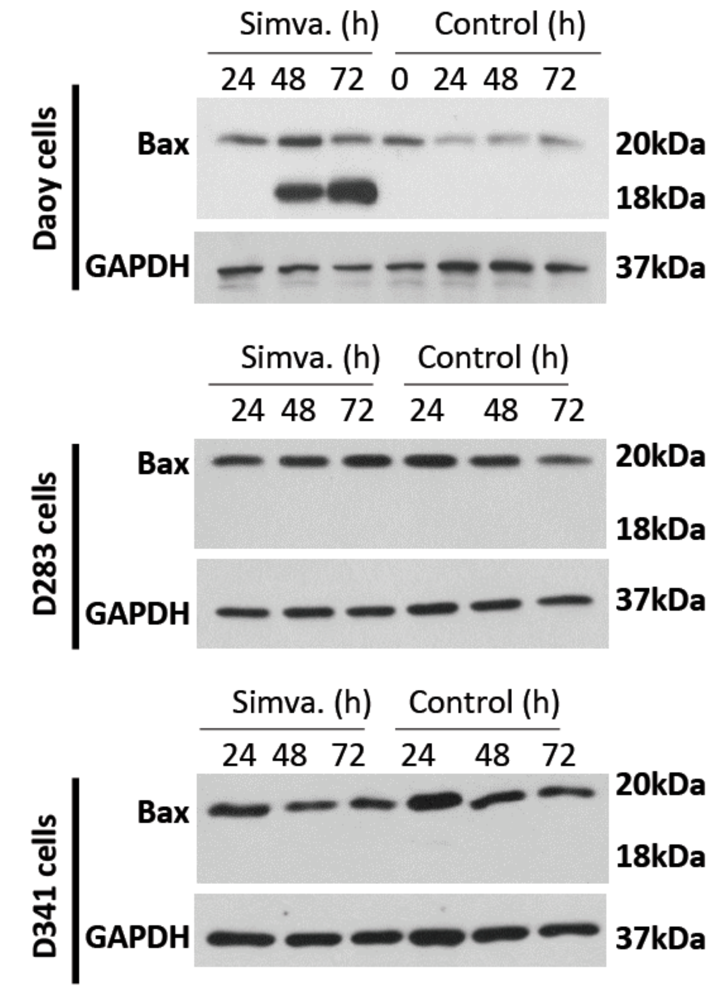

Figure S4. The continuous full length-image of Western blot signals shown in Figure 8A–C. For description of the samples, please refer to Figure legends (8A–C). Please note that for some Western blots, an additional time-point 0 has been tested. The time 0 (0 h) refers to the collected cells at the starting time for simvastatin treatments.

© 2019 by the authors. Licensee MDPI, Basel, Switzerland. This article is an open access article distributed under the terms and conditions of the Creative Commons Attribution (CC BY) license (http://creativecommons.org/licenses/by/4.0/).
